# Structural analysis of type 3 resistant starch from *Canna edulis* during *in vitro* simulated digestion and its post-digested residue impact on human gut microbiota

**DOI:** 10.3389/fnut.2024.1403497

**Published:** 2024-06-20

**Authors:** Leimengyuan Tang, Jiahui Wu, Lvbu Aga, Nan Wang, Yan Li, Houxier Li, Xueyong Wang

**Affiliations:** School of Chinese Meteria Medica, Beijing University of Chinese Medicine, Beijing, China

**Keywords:** type 3 resistant starch from *Canna edulis*, *in vitro* digestion, gut microbiota, short-chain fatty acids, bifidobacterium

## Abstract

**Introduction:**

Resistant starch (RS) has garnered attention for its health benefits, including modulating the gut microbiota and promoting the production of short-chain fatty acids (SCFAs).

**Methods:**

This study investigates structural changes of type 3 resistant starch from *Canna edulis* (CE) during *in vitro* simulated digestion and explores its health-relevant properties using healthy individuals’ fecal microbiota.

**Results:**

CE, prepared with a RS content of 59.38%, underwent a comprehensive analysis employing X-ray diffraction (XRD), fourier-transform infrared spectroscopy (FTIR), and scanning electron microscopy (SEM). During simulated digestion, XRD analysis demonstrated a significant rise in CE’s relative crystallinity from 38.92 to 49.34%. SEM illustrated the transition of CE from a smooth to a rough surface, a notable morphological shift. Post-digestion, CE was introduced into microbial fermentation. Notably, propionic acid and valeric acid levels significantly increased compared to the control group. Furthere more, beneficial *Bifidobacterium* proliferated while pathogenic *Escherichia-Shigella* was suppressed. When comparing CE to the well-known functional food fructo-oligosaccharide (FOS), CE showed a specific ability to support the growth of *Bifidobacterium* and stimulate the production of short-chain fatty acids (SCFAs) without causing lactic acid accumulation.

**Discussion:**

CE demonstrates potential as a functional health food, with implications for gut health enhancement and SCFAs production.

## 1 Introduction

Resistant starch (RS) is characterized by its resistance to enzymatic degradation in the small intestine, allowing for microbial fermentation within the colon. The resultant products of this fermentation include short-chain fatty acids (SCFAs) such as acetic acid, propionic acid, and butyric acid (1). RS can be categorized into five types (RS1 to RS5) according to its nature and granule (2). RS has been found to offer potential health benefits including the moderation of postprandial glucose and insulin levels (3), reduction of energy intake, and maintenance of glucose homeostasis (4). Retrograded resistant starch (RS3) stands out among different RS types due to its high thermal stability while cooking (as it melts at *ca.* 155°C) and its ability to resist hydrolysis by digestive enzymes, fermented by intestinal microorganisms to contribute to colonic health (5). By far, a positive correlation has been observed between amylose content and RS content in cereals. Starch with high amylose concentration is more prone to retrogradation, leading to increased RS3 levels (6).

*Canna edulis* (*C. edulis*) is a perennial herb with high medicinal value. In traditional medicine, *C. edulis* is used to prevent and treat heart disease (7). *C. edulis* tubers contain several flavonoids with excellent gastroprotective effects, reducing the ulcer index and preventing pathology in alcoholic gastritis (8). Furthermore, *C. edulis* contains many starch rhizomes, with its dried rhizomes contain 70 to 80% starch content. Extensive structural studies have been conducted on natural *C. edulis* starch (NS) (9, 10), revealing its high resistance to α-amylase (11) and a high tendency for retrogradation (12). Furthermore, there have been reports indicating that dried tubers of *C. edulis* possess a significant concentration of 30.75% amylose. This characteristic renders it a desirable raw material for the manufacture of RS3 (13). Consequently, research into RS3 preparation from *C. edulis* has been undertaken. Studies demonstrate that *C. edulis* RS3 (CE) can improve dyslipidemia and intestinal microecological health by enriching SCFAs-producing bacteria, alleviating liver steatosis, and expressing prebiotic properties (14). CE can also be used with metformin to affect the host metabolome by altering host-microbiota interactions, suggesting possible dietary fiber-drug combinations to treat type 2 diabetes (15). Current investigations into CE primarily focus on its use as a supplementary intervention or therapeutic option.

Studies have found that the long-term addition of RS fed to pigs could regulate hepatic lipid metabolism by decreasing fatty acid synthesis and increasing lipid oxidation and glycerophospholipid synthesis (16). Long-term intake of RS induces significant changes in the colonic environment, reduces damage to colonic cells, improves mucosal integrity, and reduces colonic and systemic immune reactivity (17). Therefore, it may be possible to expand the application area of CE by considering it as a functional health food.

The resistance of RS to digestion is well known. Given its carbohydrate nature, its structure and physicochemical properties do not retain their original properties after passage through the upper digestive tract. In order to emulate human dietary processes, we conducted an analysis of the structural changes observed in CE under simulated oral, gastric, and intestinal digestion. Subsequently, the post-digested CE was introduced into a microbial fermentation system using fecal samples collected from healthy human volunteers, facilitating an investigation into its fermentation attributes. The present study provides important evidence that is necessary for exploring the potential of CE as a functional food source.

## 2 Materials and methods

### 2.1 Materials

*Canna edulis* native starch was provided by Yilitai Biotechnology Co., Ltd. (Guizhou, China). Fructo-oligosaccharide (FOS) and mid-temperature α-amylase (10,000 U/g) were acquired from Solarbio Co., Ltd. (Beijing, China). Pullulanase (1,000 ASPU/mL) was purchased from Novozymes Investment Co., Ltd. (Beijing, China). An RS assay kit was obtained from Megazyme International Co., Ltd. (Wicklow, Ireland). The Milli-Q system (Millipore Corporation, MA, United States) was used to purify deionized water. Porcine pancreatic α-amylase (EC 3.2.1.1; ≥ 5 units/mg solid), porcine pepsin (EC 3.4.23.1; ≥ 250 units/mg solid), amyloglucosidase (EC 3.2.1.3; ≥ 260 U/mL liquid), and porcine pancreatin (EC 232–468-9; 8 USP solid), taurodeoxycholate (BioXtra, ≥ 97%) were purchased from Sigma-Aldrich Chemical Co. (St. Louis, MO, United States). All other chemicals and reagents used were of analytical grade.

### 2.2 Methods

#### 2.2.1 Preparation of CE samples

According to our previous work with minor modifications (18), CE was prepared as follows: 8 g of NS were weighed, dispersed in 100 mL phosphate buffer pH 4.6 (0.1 M), placed in a water bath at 100°C, and heated for 20 min.

The boiled starch solution was adjusted to 58°C, and pullulanase was added. The temperature was maintained for 12 h to achieve debranching, then mid-temperature α-amylase (1 U/g) was added for enzymolysis for 5 min. The supernatant was transferred to a boiling water bath at 100°C to inactivate enzymes, centrifuged at 4,000 rpm for 5 min, and placed at 4°C for 24 h. Finally, the recrystallized starch was washed three times at 4,000 rpm for 3 min, dried overnight at 40°C, ground, and filtered with a 200-mesh sieve for further analyses.

#### 2.2.2 *In vitro* simulated digestion

The simulated digestive process includes the oral, gastric, and intestinal phases. The preparation of simulated oral, gastric, and intestinal fluids, including the preparation of electrolyte stock solution and the addition of digestive enzymes at different stages, was performed as described (19). The experimental digestion procedure was carried out following the method of Chang et al. (20) with some adaptations. When used, the electrolyte stock solutions were diluted to 400 mL with Milli-Q-purifed water.

Oral phase: 5 mL of CE suspension (10%, w/v) was mixed with 3.5 mL simulated salivary fluid (SSF, pH = 7.0), α-amylase solution (0.5 mL, 1,500 U/mL) prepared with SSF, 25 μL of 0.3 M CaCl_2_, and 975 μL of Milli-Q-purified water. The solution was placed in a 37°C water bath and shaken for 2 min to obtain salivary digestive CE solution. We added 0.2 mL 1 M HCl to inactivate α-amylase and terminated digestion. The suspension was centrifuged at 5,000 × g for 15 min and washed in water three times. The precipitate was collected and dried overnight at 40°C to obtain a dried CE sample after saliva digestion (SSF-CE).

Gastric phase: The salivary digestive CE solution (a total volume of ~10 mL) was added with 7.5 mL simulated gastric fluid (SGF, pH = 3.0), 1.6 mL of 25,000 U/mL porcine pepsin solution prepared with SGF, 5 μL of 0.3 M CaCl_2_, 0.2 mL of 1.0 M HCl to reach pH 3.0, and 0.695 mL of Milli-Q-purified water. The mixture was placed in a 37°C water bath and shaken for 2 h to obtain gastric digestive RS solution. We added 0.2 mL 1 M NaOH to inactivate porcine pepsin. The suspension was centrifuged at 5,000 × g for 15 min and washed in water three times. The precipitate was collected and dried overnight at 40°C to obtain a dried gastric digestive CE sample (SGF-CE).

Intestinal phase: The gastric digestive CE solution (a total volume of ~20 mL) was mixed with 11 mL of simulated intestinal fluid (SIF, pH = 7.0), 5.0 mL of 800 U/mL pancreatin solution prepared with SIF, 2.5 mL of 8.0 mM cholate solution, 0.1 mL amyloglucosidase, 40 μL of 0.3 M CaCl_2_, 0.15 mL of 1.0 M NaOH to reach pH 7.0, and 1.31 mL of water. The time of contact with the enzyme was 2 h in a 37°C water bath, then 0.2 mL 1.0 M NaOH was added to inactivate pancreatin. The suspension was centrifuged at 5,000 × g for 15 min and washed in water three times. We collected the precipitate and dried it at 40°C overnight to obtain an intestinal RS sample (SIF-CE).

#### 2.2.3 RS content and starch digestibility at each digestion stage

Initially, the RS content was measured by employing the Megazyme RS assay kit in accordance with the AOAC official method 2002.02 (21, 22). Subsequently, the mass loss at different stages of digestion was determined by weighing dried samples before digestion and the different digestive processes using an analytical balance. The following equation was used to express the digestion rate.


$$\mathrm{Digestion}\;\mathrm{rate}=\frac{\mathrm{initial}\;CE\;\mathrm{mass}-\mathrm{Residual}\;\mathrm{mass}\;\mathrm{after}\;\mathrm{digestion}}{\mathrm{initial}\;CE\;\mathrm{mass}}\times 100\%$$

#### 2.2.4 Structural properties

##### 2.2.4.1 X-ray diffraction

The crystalline structure of CE samples at various digestive stages was studied using a Rigaku SmartLab-SE, and the light source was Cu-Kα radiation. Dried samples were passed through a 200-mesh sieve and placed on slides. The operation conditions were: tube voltage-40 Kv, target current-100 mA, scanning range 5–40° (2θ), and a scan rate of 2°/min. CE’s relative crystallinity (RC) and its residues during simulated digestion were quantitively estimated using MDI Jade 6.0 software according to the Nara and Komiya method (23). The RC is the ratio of the area of crystallization area to the total diffraction area.

##### 2.2.4.2 Fourier transform infrared spectrometry

Under an infrared lamp, approximately 2 mg of sample and an appropriate amount of dry potassium bromide powder were added to an agate mortar, ground thoroughly several times, and subjected to vacuum compression into transparent round tablets. The effect of water on the absorption peaks was eliminated. The pure KBr as background was collected, and then the infrared spectrum of the sample was collected at a resolution of 4 cm^−1^ with 32 scans in the wave number range of 4,000–400 cm^−1^ with a Fourier infrared spectrometer (Thermo Scientific Nicolet iS20). The spectra between 1,200 and 800 cm^−1^ were automaticlly baseline-corrected and deconvoluted employinga half-band width of 17 cm^−1^ and an enhancement factor of 1.7 using OMNIC version 8.0. The intensities for each spectrum at 995, 1022 and 1,047 cm^−1^ were obtained from the deconvoluted spectra by recording the height of the absorbance bands from the baseline. The intensity of the peak at 1047 cm^−1^ divided by that of the peak at 1022 cm^−1^ was recorded as R_1047/1022_, and R_995/1022_ was calculated using the same method (24).

##### 2.2.4.3 Scanning electron microscopy

The dried RS granules were fixed onto the sample holder using double-sided tape and coated with a gold layer using a sputter coater (Oxford Quorum SC7620). The morphology of the samples was observed using a ZEISS GeminiSEM 300 scanning electron microscope at 100×, 500×, 1 K× and 2 K×.

#### 2.2.5 Anaerobic fermentation *in vitro*

##### 2.2.5.1 Fecal collections

Fecal samples were collected from three healthy male donors (age 20–25 years) with normal BMI (18.5 kg/m^2^ < BMI < 23.9 kg/m^2^). The volunteers had been free of gastrointestinal illness for 3 months and had not taken antibiotics or bacterial preparations. Experiments as described previously (25). Fresh fecal samples were collected in sterile tubes and transferred into an anaerobic operation box. Human feces were dissolved in sterile phosphate-buffered saline (1:1, w/v) and homogenate. The fecal suspension was filtered through four layers of sterile gauze, collected, and added into a sterilized brain-heart infusion (BHI) medium at 37°C for expanded cultivation. The collection and preparation were finished within 4 h. The General Hospital of the Chinese People’s Liber-ation Army Medical Ethics Committee approved the fermentation study of human fecal microbiota on type 3 RS of *C. edulis*, registration No. S2022-379-01.

##### 2.2.5.2 Fermentation *in vitro*

The RS residues, after *in vitro* digestion, were fermented by extraction of fecal microorganisms from healthy people to study pH changes, SFCAs changes, lactate content, and gut microbiota regulation. The fermentation was carried out in an anaerobic incubator at 37°C, with gas composition of 5% CO_2_, 10% H_2_, and 85% high-purity N_2_. 100 μL the above amplified and cultured fecal suspension, 100 mg of SIF-CE was added to 10 mL of brain heart infusion broth (BHI medium, Oxoid, United Kingdom) to perform fermentation studies. The negative control group did not have any fermentation substrate added. As FOS is a macronutrient used worldwide as a food supplement or a macronutrient substitute (26), the 100 mg FOS group serves as the positive control group. At 24 h of fermentation, samples were taken from the anaerobic incubator and the reaction was stopped by 20 min of an ice bath. Samples were centrifuged at 12,000 r/min for 5 min at 4°C. The supernatant was then filtered through a 0.22 μm sterile microporous membrane and stored in a − 80°C refrigerator for SCFAs and lactate content determination. The bottom sediment was collected and used to determine the microbiota composition.

##### 2.2.5.3 Determination of pH value

The SIF-CE, FOS and the control group fermentation products were collected at 0, 4, 12, 24 h. These fermentation products were centrifuged at 12,000 r/min for 5 min at 4°C. The supernatant was filtered through a 0.22 μm sterile microporous membrane and transferred into screw tubes. A pH meter measured the pH values (FE28-Standard, Mettler Toledo Instruments Co. Ltd., Shanghai, China).

##### 2.2.5.4 Determination of SCFA content

SCFAs concentration in the three groups were measured according to the Zhang et al. with some modifications (27). SCFAs were determined using 7820A-5977B gas chromatograph-mass spectrometer (Agilent Technologies Inc. CA, United States) fitted with a DB-FFAP (30 m × 0.25 mm × 0.25 μm) and electron ionization. The initial temperature of the column was set at 70°C and held for 5 min. Then, the temperature increased to 100°C at 6°C/min. The MS data was acquired in full scan mode from m/z 30–550 with the injection temperature set as 260°C. SCFAs contents were calculated according to the calibration curves of respective standards (Supplementary Table S1).

##### 2.2.5.5 Determination of lactate content

Based on the supplier’s instructions, the lactic acid contents in the SIF-CE, FOS and the control group were measured with a lactic acid assay kit (Nanjing Jiancheng Biotechnology Co. Ltd., Nanjing, China).

##### 2.2.5.6 DNA extraction and 16S rRNA sequencing

The QIAamp Fast DNA Stool Mini Kit was used to extract total bacterial genomic DNA samples from fecal samples. The DNA purity and concentration was measured on a Nanodrop 2000 spectrophotometer (Thermo Scientific, United States). The polymerase chain reaction was used to amplify the V3-V4 region of bacterial 16S rDNA using the forward primer 338F (5’-ACTCCTACGGGAGGCAGCA-3′) and reverse primer 806R (5’-GGACTACHVGGGTWTCTAAT-3′) (28). Three replicates of each group were used for data analysis, and some of the data were analyzed on Meguiar’s BioCloud platform.^1^

#### 2.2.6 Statistical analysis

Results were expressed as mean ± standard deviation for three replicates. Statistical differences were determined using GraphPad Prism 9.0 (Inc., California, United States). Differences between groups were analyzed using a one-way analysis of variance, with differences considered statistically significant at *p* < 0.05.

## 3 Results

### 3.1 RS content and digestibility

The RS content of CE was 59.38% (Table 1) After a three-stage digestion (oral-gastric-intestinal), the final digestibility of CE was 33.47%. Notably, the digestibility results exhibited a correlation with the RS content. About 66.53% of the mass of CE entered the fermentation stage, and CE showed good resistance to enzymatic and ionic attacks during the digestion stage. CE showed good resistance to digestion in the oral cavity at only 4.84%, and digestion occurred primarily in simulated gastric and small intestinal fluids. Pepsin hydrolyses hydrophobic amino acids (29); therefore, the weight loss of CE during the gastric digestion phase may be due to the hydrolysis of some soluble starch under acidic conditions, while the intestinal phase may be the result of a combination of enzymes as well as ionic action.

**Table 1:** RS content of CE and its digestibility in oral, gastric and intestinal fluid phases.

Sample	RS content	digestibility (%)		
		Oral phase	Gastric phase	Intestinal phase
CE	59.38 ± 0.71	4.84 ± 0.78	15.46 ± 1.75	33.47 ± 2.19

### 3.2 Structural characterization

#### 3.2.1 X-ray pattern and relative crystallinity

**Figure 1:**
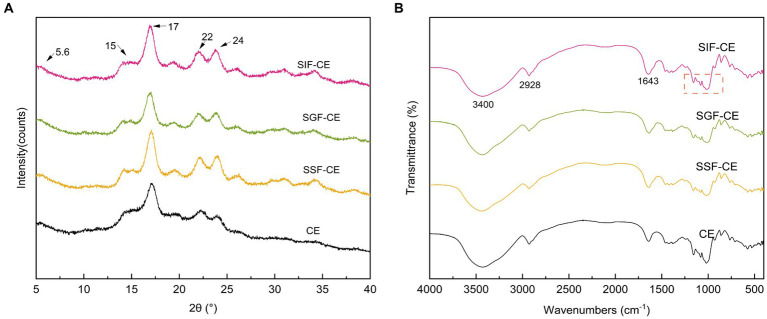
**(A)** XRD patterns of CE and its residues of different simulated digestive stages. **(B)** FTIR spectra of CE and its residues of different simulated digestive stages. (SSF-CE, dried CE sample after saliva digestion; SGF-CE, dried CE sample after gastric digestion; SIF-CE, dried CE sample after intestinal digestion).

The XRD pattern of RS includes the crystalline and amorphous regions (30). The crystalline region is composed of stacked straight-chain starch double helixes, while the amorphous region is composed of disordered starch. As shown in Figure 1A, the CE samples at various digestive stages showed a typical B-crystalline morphology. Diffraction peaks appeared at 5.6°, 15°, 17°, 22°, and 24° (2θ) (31).

The level of crystallinity showed an upward trend with the progression of digestion (Table 2). The RC of CE was determined to be 38.92%. Following *in vitro* digestion with different digestive enzymes, the RC was 41.70% for SSF-CE, 46.01% for SGF-CE, and 49.34% for SIF-CE, separately. The depolymerization of starch structure usually occurs in the amorphous regions. The structural changes in CE samples manifest as the action of digestive enzymes and various ions on the amorphous regions, leading to a significant increase in crystallinity. Nevertheless, there was no substantial effect on the crystal type.

**Table 2:** RC, DO, and DD of CE and its residues at different simulated *in vitro* digestion.

Sample	RC	R_1047/1022_	R_995/1022_
CE	38.92 ± 0.04^a^	1.56 ± 0.04	0.96 ± 0.13
SSF-CE	41.70 ± 0.16^b^	2.43 ± 0.46	1.70 ± 0.32
SGF-CE	46.01 ± 0.56^c^	2.27 ± 0.65	1.77 ± 0.60
SIF-CE	49.34 ± 0.47^d^	2.81 ± 0.72	1.86 ± 0.74

Means ± standard deviations are calculated from repeated measurements, and different letters in the same column indicate a significant difference (*p* < 0.05).

#### 3.2.2 Fourier-transform infrared spectroscopy spectrum of CE

Figure 1B showed the FTIR spectra between wave number 4000–400 cm^−1^ of CE and its corresponding digestive samples. All samples exhibited a broad absorption bands at 3400 cm^−1^ which can be attributed to O-H stretching vibration of starch (32). The absorption peaks at 2928 cm^−1^ and 1643 cm^−1^ are ascribed to C-H stretching (33) and O-H bending vibration (34), respectively. The infrared spectra showed that no peaks disappeared or new peaks were generated after the various stages of digestion or enzymatic digestion, suggesting that no new covalent bonds or functional groups were generated after the *in vitro* digestion of CE.

The infrared peak range of 1200–800 cm^−1^ (Figure 1B, red dotted coil) was deconvoluted to analyze the short-range order of starch. The absorbance of starch at 1047 cm^−1^ and 1022 cm^−1^ reflects the ordered crystalline structure as well as the amorphous structure of starch (35). The absorbance ratio of 1047/1022 (R_1047/1022_) can be used to indicate the degree of ordered structure (DO) values of starch crystals. The absorbance ratio of 995/1022 cm^−1^ (R_995/1022_) for starch reflects the degree of double helix (DD) values between starch molecules (36). The DO and DD of CE and its digestive samples are shown in Table 2. These calculations revealed that DO and the DD of CE did not differ significantly between the groups after the action of different digestive enzymes; there was an upward trend, coinciding with the XRD pattern.

#### 3.2.3 Scanning electron microscopy and morphological characteristics

**Figure 2:**
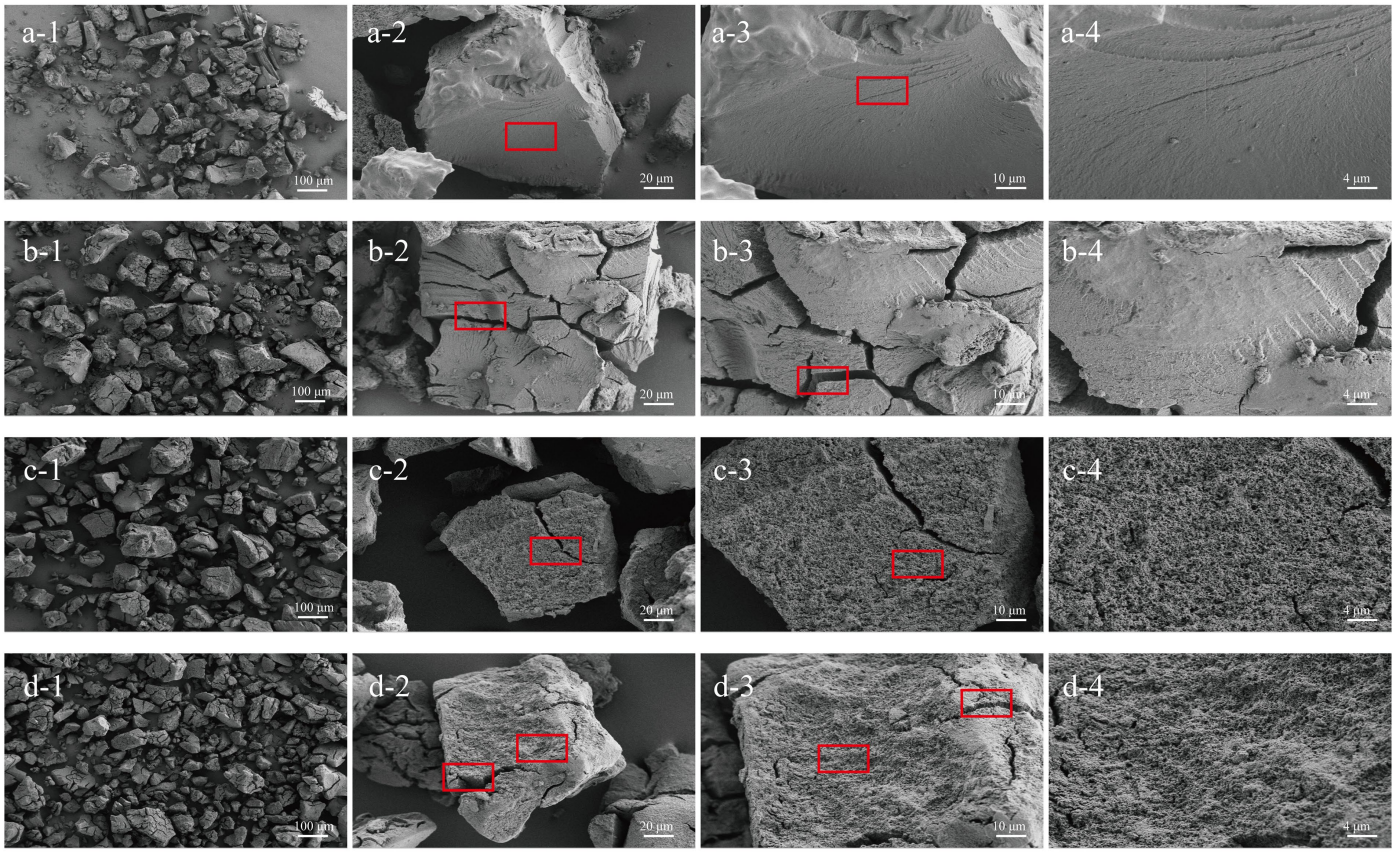
SEM of CE at various simulated *in vitro* digestive stages. CE (a1-a4), oral phase (b1-b4), gastric phase (c1-c4) and intestinal phase (d1-d4) at various magnifications (100 ×, 500 ×, 1 K × and 2 K ×).

**Figure 3:**
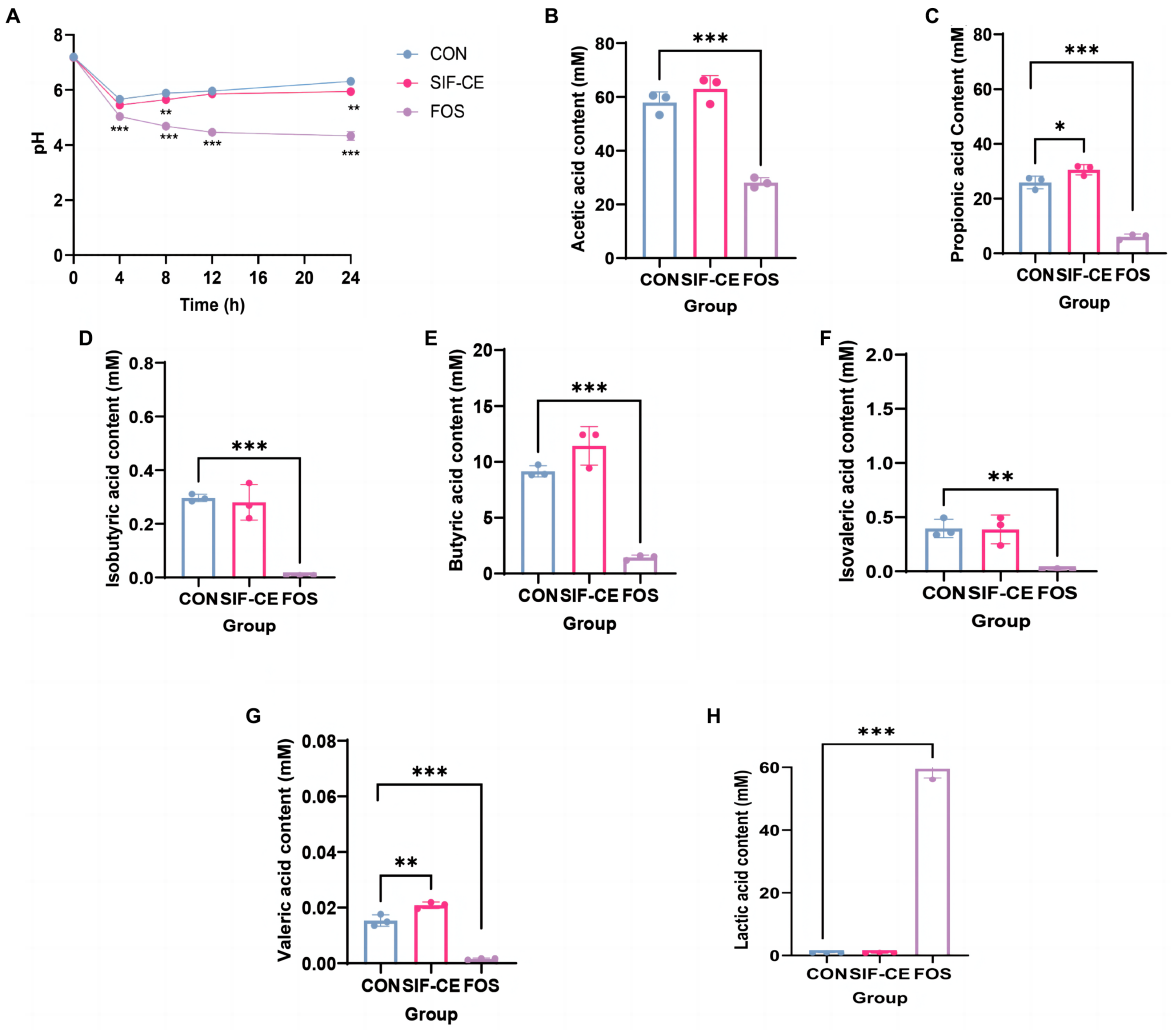
Monitoring pH dynamics, final short-chain fatty acid changes, and lactic acid content in healthy human feces for 24 h after *in vitro* simulated digestion of SIF-CE. **(A)** pH values during *in vitro* fermentation. **(B)** Acetate, **(C)** propionate, **(D)** Isobutyric acid, **(E)** butyric acid, **(F)** Isovaleric acid, **(G)** valeric acid and **(H)** Lactic acid production produced during *in vitro* fermentation. Compared to the control group, *p < 0.05, **p < 0.01, ***p < 0.0001.

As shown in Figure 2, a-3, CE exhibited a smooth surface and a compact structure, which is likely due to the retrogradation of amylose chains. Cracks began to appear on the surface of CE particles at the oral phase, indicating the initial mechanical breakdown of the particles during chewing (37). Despite these cracks, the surface remained relatively smooth, suggesting limited enzymatic activity at this stage (Figure 2, b-3). During the gastric phase, the surface of the CE granules developed honeycomb pores (Figure 2, c-3), possibly as a result of the breakdown of the amorphous portions of the resistant starch in the acidic environment. The final digestion product, SIF-CE, exhibited a rough surface with large cracks and a homogeneous honeycomb structure. This morphology indicated extensive enzymatic degradation. However, the 500 × images (Figure 2, a-2; b-2; c-2; d-2) showed that the size of the CE particles did not change significantly at different stages of digestion.

### 3.3 Anaerobic fermentation

#### 3.3.1 pH change during *in vitro* fermentation

SIF-CE was subjected to enteric bacterial fermentation experiments in healthy people using a BHI medium at a 1% addition rate. Initially, the pH of all three groups was approximately 7.2 (Figure 3A). However, within the first 4 h, there was a sharp decline in pH observed in the control, SIF-CE, and FOS groups. This finding can be attributed to the rapid consumption of nutrients within the medium by fecal microbiota, resulting in their extensive proliferation and subsequent acid production.

During the anaerobic fermentation process, a noticeable deviation in pH levels emerged between the SIF-CE and control groups after 8 h. Subsequently, the pH levels in both the control and SIF-CE groups reached 6.31 and 5.95 over time, and the SIF-CE group had a significantly lower pH than the control group. The pH of the FOS group continued decreasing until it stabilized at around 4.33 at 24 h. These findings suggested that the consumption of CE and FOS led to a considerable reduction of pH after fermentation by enteric bacteria.

#### 3.3.2 Short-chain fatty acids generated by *in vitro* fermentation and their changes

The SCFAs levels in the supernatants after microbial fermentation of feces in a healthy population were measured. Figure 3 showed, six SCFAs were detected in all three groups after 24 h of fermentation, including acetic, propionic, isobutyric, butyric, isovaleric, and valeric acids. Notably, the introduction of SIF-CE for fermentation led to a significant increase in the levels of propionic and valeric acids, as depicted in Figures 3C,G. Conversely, the FOS group exhibited significantly lower SCFAs levels in the FOS group compared to the control group.

SCFAs levels in the fermentation supernatants supplemented with SIF-CE were significantly higher than in the FOS group. SIF-CE was more potent than FOS in promoting SCFAs production.

#### 3.3.3 Changes in the content of lactic acid

The degradation of carbohydrates by intestinal microorganisms produces organic acids, and lactic acid is a primary organic acid. Therefore, we examined the lactic acid content in the supernatants after 24 h of fermentation. Figure 3H reveals that the lactic acid content in the FOS group was significantly higher than the control group by approximately 60-fold. Fecal microorganisms use FOS to produce large amounts of lactic acid. There was no significant change in lactate content in the SIF-CE group compared with the control group. These findings suggest that the pH reduction of the fermentation system caused by SIF-CE and FOS is achieved by different mechanisms, the former by promoting the content of SCFAs and the latter by increasing the lactic acid content to achieve low pH.

### 3.4 Regulation of the composition of the intestinal microbiota

#### 3.4.1 Changes in the structure of the gut microbiota

**Figure 4:**
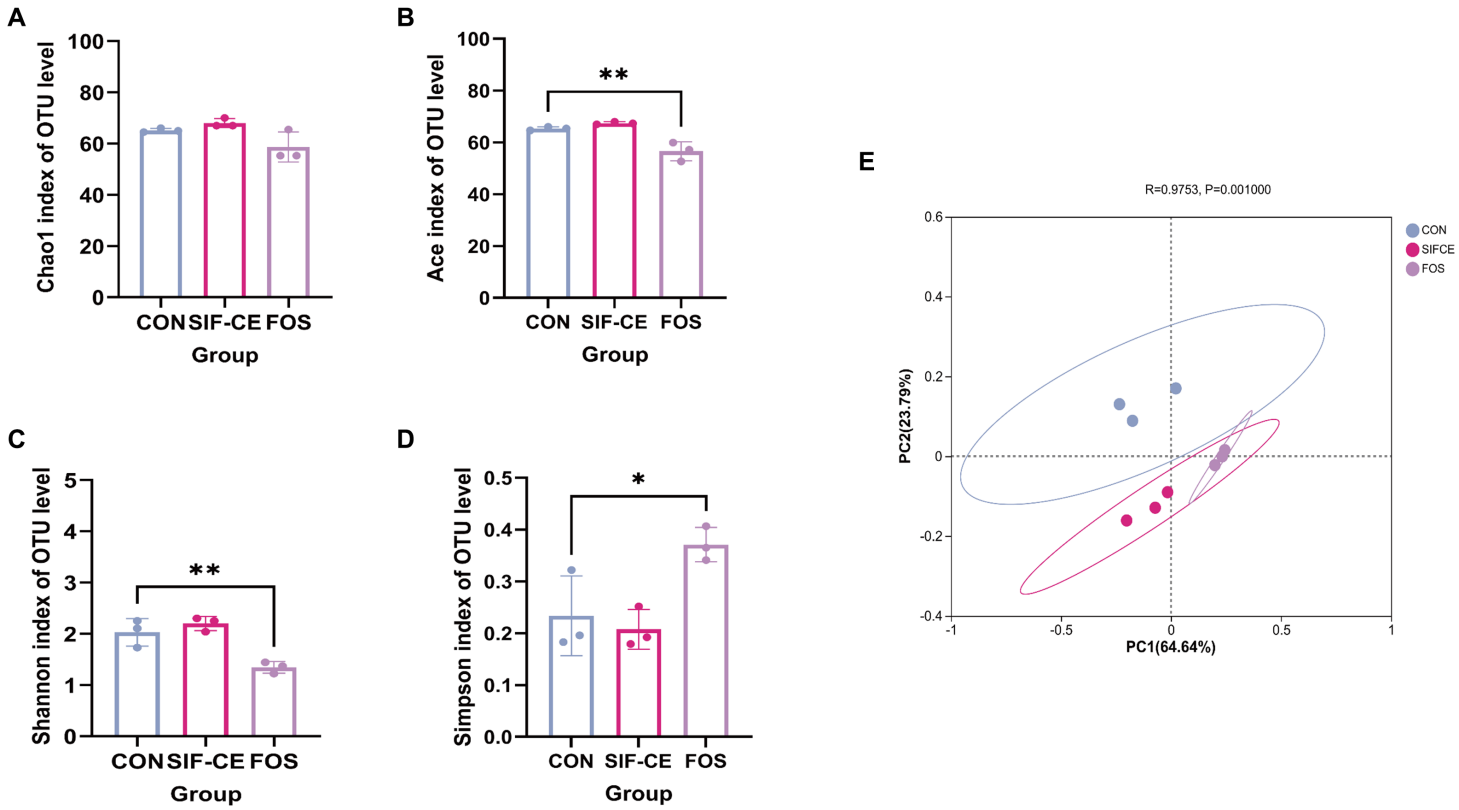
α-diversity **(A–D)** and β-diversity **(E)** of the intestinal microbiota after 24 h of fermentation with SIF-CE.

Gut microbiota diversity analysis used 97% sequence similarity genes as the operational taxonomic unit delineation threshold. Alpha diversity indexes measure intra-group colony diversity from various perspectives. The alpha diversity index, Chao1 index, and Ace index are commonly used to characterize the species richness of a colony. The Shannon and Simpson indexes combine the richness and evenness of the community. Combining all alpha diversity indexes (Figures 4A–D), there was no significant difference in the effect of SIF-CE addition on the internal diversity of the fecal microbial community and no significant effect on species richness compared to the control group. In contrast, FOS had significantly lower Ace, Shannon’s index (*p* < 0.01), and Simpson’s index (*p* < 0.05), suggesting that FOS significantly reduced species richness and diversity.

The β-diversity index focuses on the differences between samples, using primary coordinates analysis to assess the β-diversity of fecal microorganisms after 24 h fermentation. Figure 4E shows that the groups are separated. The first (PC1) and second principal components (PC2) explained 64.64 and 23.79% of the total variables, respectively. Combined with the Anisom analysis, the differences in intestinal microbiota between the groups were statistically significant (*p* < 0.01).

#### 3.4.2 Bacterial species composition analysis

**Figure 5:**
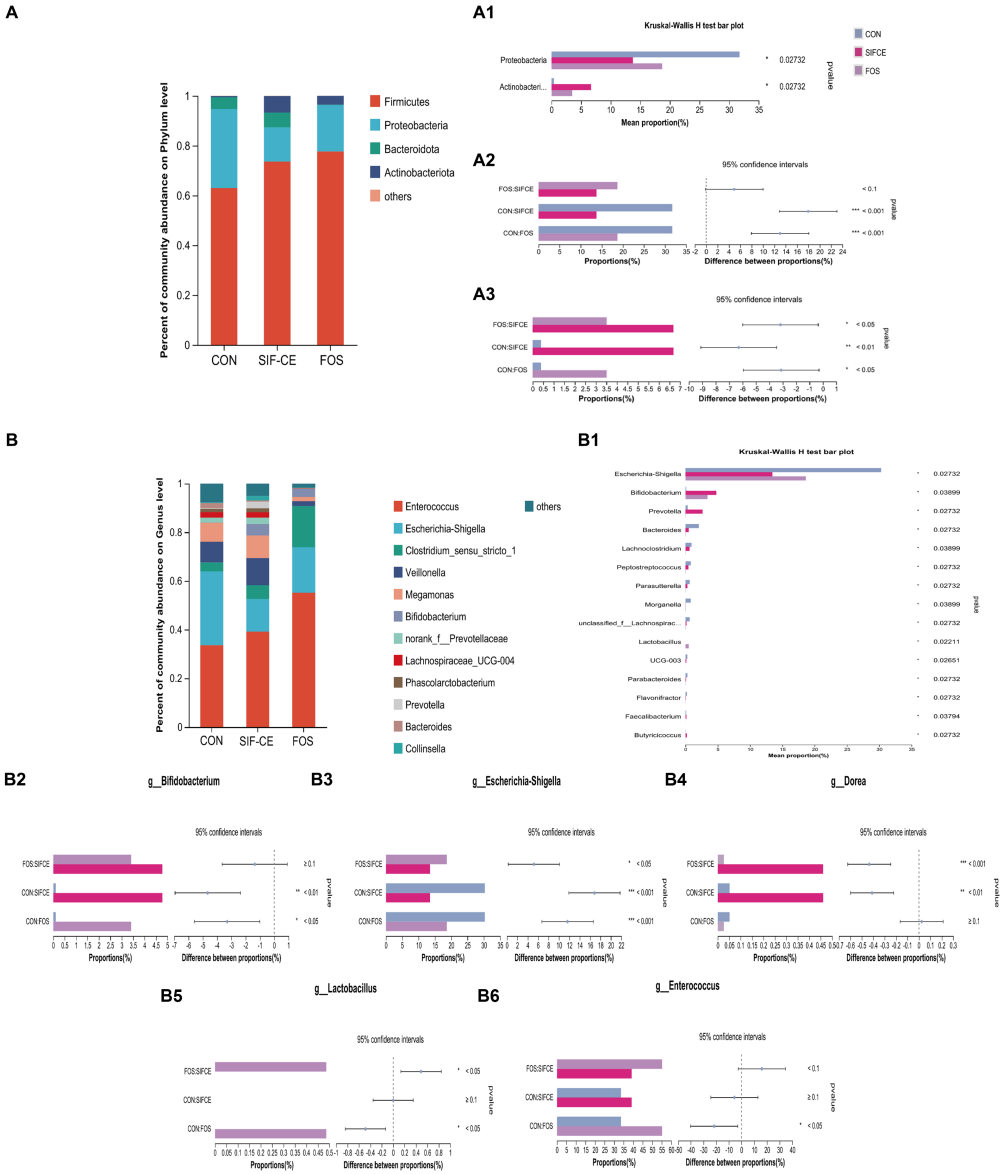
Microbial composition at the phylum level. **(A)** A1 Phylum with significant differences within the three groups; A2. Proteobacteria proportion and significant differences within groups; A3. Actinobacteriota proportion and significant differences within groups and at the genus level. **(B)** B1 Genus with significant differences between the three groups; B2. *Bifidobacterium* proportion and significant differences within groups; B3. *Escherichia-Shigella* proportion and significant differences within groups; B4. *Dorea* proportion and significant differences within groups; B5. *Lactobacillus* proportion and significant differences within groups; B6. *Enterococcus* proportion and significant differences within groups after 24 h of fermentation with SIF-CE (*p < 0.05, **p < 0.01, ***p < 0.001).

To investigate the differences in community structure among different fermentation groups, the changes in species abundance at the phylum level are shown in Figure 5A. The three groups on the phylum level are mainly composed of Firmicutes, Proteobacteria, Bacteroidota, and Actinobacteriota. The Kruskal-Wallis test showed significant differences in abundance between the three groups found for *Proteobacteria* and *Actinobacteriota*, (Figure 5, A1). Compared with the control group, the *Proteobacteria* content in the SIF and FOS groups decreased significantly after 24 h of fermentation (Figure 5, A2). SIF-CE showed the same ability as the FOS group to upregulate *Actinobacteriota* significantly, and the amount of *Actinobacteriota* was significantly higher in the SIF-CE group than in the FOS group (Figure 5, A3).

At the genus level, the Kruskal-Wallis test showed that the relative abundance of 15 genus was statistically significant (Figure 5, B1). In the FOS group, the abundance of *Anaerostipes*, *Holdemania*, and *Coprobacillus* was significantly decreased to a proportion of approximately 0% (*p* < 0.01). These genus showed the same trend of being downregulated in the SIF-CE group and were also significant (*p* < 0.05).

The well-known probiotics such as *Bifidobacterium* and *Lactobacillus*, which can produce lactic acid (38) were studied. As shown in Figures 5, B2, B5, in the control and SIF-CE groups, the abundance of *Lactobacillus* was minimal and barely observable on the bar graph; however, its abundance increased significantly and significantly in the FOS group. The abundance of *Bifidobacterium* was 0.108% in the control group and 4.775% in the SIF-CE group, approximately 44 times more in the SIF-CE group than in the control group. FOS also showed an upregulation of *Bifidobacterium.*

In addition to these results, we found that the proportion of *Enterococcus* (Figure 5, B6) abundance in the FOS group was large and significant relative to the control group, and *Dorea* (Figure 5, B4) was significantly upregulated in the SIF-CE group. *Dorea* is essential in non-alcoholic steatohepatitis and is one of the primary regulators of microbiota-dependent protective phenotypes in mitochondrial microbiota interactions (39). *Enterococcus* is usually capable of producing lactic acid (40). The SIF-CE and FOS groups showed the same ability to downregulate *Escherichia-Shigella* (Figure 5, B3).

## 4 Discussion

In this study, our primary objective was to explore the feasibility of harnessing CE as a potential candidate for the development of a functional health product. Following the modification of a previous preparation method to produce CE with a measured RS content of 59.38%, we conducted an *in vitro* simulated digestion study to evaluate CE’s resistance to specific digestive enzymes. By inhibiting the digestion of starch, RS results in a delayed release of glucose, which contributes to reduced glycemic and insulinemic responses (41). Our investigation revealed that CE exhibited notable resistance to the action of digestive enzymes, as evidenced by the preservation of CE mass, consistent with its initial RS content, following the final digestion phase.

The XRD study of CE at various digestive stages revealed a noteworthy increase in the RC of post-digested CE residue. This rearrangements of RS may also occur during digestion, promoting crystal formation and growth (42). Consistent with RC results are the changes in the double helicity of CE during digestion, although these changes were not statistically significant.

The surface morphology of CE was analyzed. Notably, CE showed a smooth surface and dense structure with stable size during digestion, which contributed to its resistance against enzymatic breakdown during digestion. Studies showed that in superior starch granule structures, α-amylase (with a radius of about 3 nm) cannot pass through starch granules lacking pores, and its enzymatic capacity is significantly inhibited (43, 44). Furthermore, research has indicated that a decrease in starch particle size increases the surface area exposed to digestive enzymes, consequently accelerating the digestion rate (45). Notably, CE underwent a significant morphological alteration, exhibiting a rough surface with numerous perforations. Studies have shown that these rough surfaces on RS granules facilitate the proliferation of *Bifidobacteria* (46). This rough surface characteristic is critical for comprehending the fermentative behavior of CE in fecal microbiota.

We subjected the residual CE to simulate *in vitro* fermentation studies. By monitoring the pH levels within the fermentation system, we observed significant reductions induced by both SIF-CE and FOS. SIF-CE ended up at a stable pH of 5.94, and FOS ended up at 4.33. A lower intestinal pH environment can inhibit the invasion and growth of potentially pathogenic bacteria (47), directly affect the microbiota (48), and have a beneficial effect on the absorption of minerals in the intestinal tract (49).

SCFAs are the key components that often decrease the pH of the system following fermentation and have a crucial role in maintaining the health of the gastrointestinal tract. The SIF-CE group had markedly elevated levels of propionic and valeric acids compared to the control group. Propionic acid has beneficial health activities, including lowering cholesterol concentrations, reducing fat storage, and inhibiting gluconeogenesis (50). Likewise, valeric acid protects dopamine neurons by inhibiting oxidative stress and regulating autophagic pathways. It also has an antihypertensive effect by causing vasodilation (51, 52). These findings indicate that there are intriguing areas for further investigation, highlighting the potential of CE as a functional health food option, especially for addressing lipid metabolism and brain health.

The reduced concentrations of SCFAs in the FOS group, despite its low pH measurements, prompted further investigation. It was seen that the FOS group displayed a substantial increase in lactate levels, with a recorded value of 59.60 mM (Figure 3H). This discovery is consistent with previous studies that have shown that at a pH level of 5.2, the synthesis of lactate is maintained while its consumption is inhibited, resulting in the accumulation of lactate (50). Consuming FOS can result in the buildup of lactic acid and affect the survival of bacteria that metabolize lactate, as well as the levels of SCFAs. The lactic acid content is the key element that influences the pH of the FOS system, based on the combined results of pH and SCFAs (53). When directly compared, it can be deduced that CE and FOS have different effects on the regulation of fermented system. When directly compared, it can be deduced that CE and FOS have different effects on the regulation of fermented system. The primary determinant of pH in the context of CE is the presence of SCFAs, while the primary factor influencing pH regulation in relation to FOS is the level of lactic acid concentration.

Microbiota composition in the gut is a key determinant of gut health. We explored how the introduction of CE and FOS affected the gut microbiota. At the genus level, the FOS group exhibited a significant pro-proliferative capacity for *Bifidobacterium* (Figure 5, B2) and *Lactobacillus* (Figure 5, B5). This proliferation aligns with previous studies (54). *Bifidobacterium* and *Lactobacillus* can produce lactic acid by fermentation using prebiotics. Through cross-feeding between species, lactic acid can be metabolized into the production of SCFAs such as acetic, propionic, and butyric acids through cross-feeding between species (55). Under normal conditions, lactic acid is utilized by lactic acid-utilizing bacteria in the intestine and does not lead to accumulation. However, some lactic acid-utilizing species are sensitive to low pH, and lactic acid-producing bacteria are resistant to the low pH environment; as a result, lactic acid can accumulate in low pH environments (56). The accumulation of lactic acid leads to a rapid shift in the overall microbial community composition from a community dominated by Bacteroidota and Firmicutes to a community dominated by *Lactobacillus* and *Proteobacteria* (57). Prebiotics may have limited the abundance of some genera through two mechanisms: firstly, by promoting the growth of specific species with specialized digestive functions, thereby creating competition. Another approach involves reducing the pH of the intestines by the fermentation of prebiotics, leading to the synthesis of organic acids, specifically SCFAs. This phenomenon may explain the high lactate content and the significant decrease in alpha diversity in the FOS group (Figure 5A). The lactic acid produced by *Bifidobacterium* proliferation may increase the content of SCFAs in the system through cross-feeding in SIF-CE group. FOS promoted *Lactobacillus* and *Bifidobacterium*, which causes a rapid decrease in system pH, leading to the accumulation of lactic acid and downregulation of pH-sensitive bacterial genus in the system, resulting in a decrease in the content of SCFAs produced by metabolism.

Conversely, SIF-CE did not significantly affect the alpha diversity of the fecal microbiota. However, there was a noteworthy increase in the abundance of *Bifidobacterium*, a commonly used probiotic, which has essential pharmacological effects in reducing symptomatic abdominal pain in irritable bowel patients and as an anti-inflammatory (58, 59). It is worth noting that structural changes in CE after digestion, such as rough surfaces, high crystallinity, and a stable double helix structure, may promote the selective proliferation of *Bifidobacterium* (60). Furthermore, the type 3 resistant starch of B crystals present in CE has been reported to stimulate the growth of *Bifidobacterium* (61). Remarkably, SIF-CE and FOS exhibited the same inhibition of *Escherichia-Shigella* growth, which is considered harmful because it has been reported to be associated with irritable bowel syndrome.

In summary, CE exhibits several advantages in promoting gut health and modulating the microbiota. It significantly elevates levels of SCFAs, such as propionic and valeric acids, without causing lactic acid accumulation. Unlike FOS, which can disrupt pH balance and decrease alpha diversity, CE maintains these aspects while selectively promoting the proliferation of *Bifidobacterium*, a well-known probiotic. These findings indicate that CE holds great promise as a candidate for functional health foods.

## 5 Conclusion

CE undergoes a structural change after *in vitro* simulated digestion, which are essential for its bacterial fermentation in the intestine. After fermentation by fecal microorganisms, CE significantly upregulates propionic and valeric acid content and downregulates pH, increasing the abundance of beneficial genera, especially *Bifidobacterium*, and downregulating pathogenic genera such as *Escherichia-Shigella* in healthy people without affecting microbial diversity. These functions are essential for maintaining a healthy intestinal tract and have significant potential for development as a functional food for healthcare.

## Data availability statement

The original contributions presented in the study are included in the article/Supplementary material, further inquiries can be directed to the corresponding author.

## Author contributions

LT: Conceptualization, Data curation, Writing – original draft, Writing – review & editing, Project administration, Software. JW: Methodology, Validation, Writing – original draft, Writing – review & editing, Formal analysis, Software. LA: Formal analysis, Software, Writing – review & editing. NW: Conceptualization, Investigation, Writing – review & editing. YL: Data curation, Investigation, Supervision, Writing – review & editing. HL: Formal analysis, Validation, Writing – review & editing. XW: Funding acquisition, Resources, Writing – review & editing.

## Footnotes

^1^https://cloud.majorbio.com
