# Supplementary material for: Structural analysis of type 3 resistant starch from Canna edulis during in vitro simulated digestion and its post-digested residue impact on human gut microbiota
Source: Front Nutr. 2024 Jun 20;11:1403497. doi: 10.3389/fnut.2024.1403497 (PMC11223600; doi:10.3389/fnut.2024.1403497)
Supplement: Supplementary file 1 [file Table_1.DOCX]

Table S1. Analytical parameters for GC-MS analysis of SCFAs.

| SCFAs | Retention time (min) | Correlation coefficients | Calibration curves |
| --- | --- | --- | --- |
| Acetic acid | 4.218 | 0.9996 | y=14350.543501* x |
| Propionic acid | 5.137 | 0.9996 | y = 6892.790554 * x |
| Isobutyric acid | 5.467 | 0.9989 | y = 22410.247794*x |
| Butyric acid | 6.268 | 0.9997 | y = 9817.993549 * x |
| Isovaleric acid | 6.85 | 0.9994 | y=11117.934665*x |
| Valeric acid | 7.885 | 0.9992 | y=31601.496102 * x |
